# Mechanical and Environmental Performance of Concrete Incorporating Post-Consumer Plastics and E-Waste

**DOI:** 10.3390/ma19061259

**Published:** 2026-03-23

**Authors:** Madiha Ammari, Halil Sezen, Jose Castro

**Affiliations:** 1 Department of Civil and Environmental Engineering, Morgan State University, Baltimore, MD 21251, USA; 2 Department of Civil, Environmental and Geodetic Engineering, The Ohio State University, Columbus, OH 43210, USA; sezen.1@osu.edu; 3 Department of Integrated Systems Engineering, The Ohio State University, Columbus, OH 43210, USA; castro.38@osu.edu

**Keywords:** plastic, polypropylene, printed circuit boards, cement, aggregate, concrete, compressive strength, flexural behavior, life cycle assessment, waste valorization

## Abstract

A significant portion of plastic products is not accepted by curbside recycling companies and goes to landfills or incineration, causing an adverse impact on the environment. This study investigated the effects of utilizing post-consumer plastic and e-waste in concrete. A plastic product made of thermoplastic polypropylene (PP) was ground into fine particles and used for 10% volumetric replacement of sand, while bare printed circuit boards (PCBs) were pulverized into powder and used for 10% cement replacement by mass. This study introduces a unique utilization of grounded powder PCBs by partially replacing cement in concrete. Furthermore, reinforced concrete beams with the replacements were constructed and tested under flexure for structural behavior evaluation. The results of this study show an average of 11% reduction in both the compressive strength of concrete and the maximum load capacity of the beams incorporating plastic products. A life cycle assessment study was conducted using a functional unit of 1.0 cubic yard concrete production. The system boundary for the environmental assessment of the concrete in this study includes only the production phase, which is from the cradle to the end gate of the ready-mix concrete plant. The environmental impact estimation of a 10% reduction in constituents of concrete showed a 10% reduction in most LCA measures where cement was replaced compared to a 1% effect for the fine aggregate replacement.

## 1. Introduction

Plastic has wide applications because it is easy to form and shape, it is a lightweight material, and it is water- and chemical-resistant. While it has a long life, most plastics are utilized for packaging purposes and used one time and then disposed of in the environment. In comparison, plastics used in electronics, including printed circuit boards, have a longer service life, and their disposal processes introduce environmental issues.

There are two main types of plastics: thermoplastics and thermosetting polymers. The main difference between the two is that thermoplastics can be heated and remolded several times because thermoplastics melt when heated and solidify as they cool down. On the other hand, thermosetting plastics can be set, shaped, and molded just once and harden into a permanent shape. The ability to melt thermoplastics once heated makes it a material that is applicable for recycling; however, the permanent structure of the thermosets complicates its recycling process. Since thermal reprocessing does not apply to thermosets, they are usually recycled as fillers [1].

Thermoplastics are classified into seven different types depending on their chemical compound: Type 1: Polyethylene terephthalate (PET), Type 2: High-Density Polyethylene (HDPE), Type 3: Polyvinyl Chloride (PVC), Type 4: Low-Density Polyethylene (LDPE), Type 5: Polypropylene (PP), Type 6: Polystyrene (PS), and Type 7: miscellaneous plastics. Miscellaneous plastics include all other types of plastics, such as polycarbonate, polylactide, acrylonitrile butadiene, styrene, fiberglass reinforced plastic, and nylon [1].

In 2018, a total of 292 million tons of municipal solid waste (MSW) was generated in the U.S., and plastics made up 12.2% of the total waste, with only 4.5% of the total plastic waste recycled [2]. In Europe, the highest type of plastic in demand is PP, followed by LDPE, and then PVC, HDPE, PS, and PET. Types 1 to Type 6 are thermoplastics, which are theoretically recyclable, while some of the plastic in Type 7 are thermosets, including some e-waste such as epoxy resin in electronic printed circuit boards (PCB)s [3]. In the U.S., the plastic type with the highest recycling rate is PET, approximately 20%, followed by HDPE and LDPE. Other plastic types, including PVC, PP, and PS, which contribute to approximately 35% of the total mass in MSW, are not recycled [4].

Considerable research studies varied in incorporating different types of plastic in concrete and conducted their research by recycling post-consumer plastic products (e.g., [5,6,7,8,9,10,11,12,13]). In such studies, plastic was either used to replace aggregate or used as fibers. They mainly concentrated on investigating the effect of using plastic waste on the different mechanical properties and durability of the produced concrete. However, the research lacks further studies to focus on evaluating the structural performance of reinforced concrete elements incorporating plastics.

In comparison, the printed circuit board or PCB waste represents approximately 3% of the total mass of the e-waste material stream. Due to the diversity and complexity of waste PCBs in terms of shape, size, type, components, and composition, the recycling process for this waste is difficult. In addition, the presence of materials with different characteristics (plastics, ceramics, and metals) in this waste leads to difficult liberation and separation [14]. However, if appropriately recycled, it is useful due to its high content of metals that can reach up to 30% of the PCB mass. Copper alone was found to constitute up to 20% of the PCBs [15].

In PCB recycling facilities, precious metals such as Au, Pd, Ag, and Cu are separated and refined from crushed PCB waste. The non-metallic material in PCB waste makes up almost 70% of its mass [16]. The non-metallic materials which consist of plastic resin, fiberglass, and ceramic are then disposed of by combustion or landfilling.

The metal fraction refining process in PCB waste is usually employed using hydrometallurgical processes. In this process, the metal contents are dissolved into leaching solutions consisting of strong acids such as sulfuric acid or hydrochloric acid [17]. This process leaves bare PCBs behind, which are composed of layers of copper sheets strengthened by the substrate of glass-fiber-reinforced epoxy resin. Later, if the initial investigation determines valuable traces of precious metals, in addition to rich copper substrate, the PCBs are further processed by using hammer mills to crush and grind the bare PCB into particles of a maximum size of 2 mm. A density separation method, such as shaking tables, is then used to separate metals from non-metallic components of the PCBs. This research study aims to utilize the non-metallic components resulting from the metal-refining process of bare PCBs.

In the U.S., most generated e-waste is either unofficially exported, discarded in landfills, or incinerated. Worldwide, e-waste is the largest category of illegally traded hazardous solid waste [18]. Recycling e-waste in some countries does not follow health regulations, resulting in a significant risk of toxic exposure to recyclers. Several million metric tons of electronic waste (e-waste) are yearly discarded worldwide [19].

Unfortunately, using concrete as a construction material has a negative impact on the environment, which is associated with the production of each of its constituents. In the cement industry, the clinker production process contributes 8% of the total worldwide emissions of CO_2_ [20]. In addition, sand mining may harm different animal habitats, and sand exploitation causes environmental damage [21]. Reducing the amount of natural sand used in the construction sector should help reduce the adverse environmental impact of concrete.

While considerable research studies varied in incorporating different types of plastic in concrete, limited research studies concentrated on using PCB waste in concrete. In a study conducted in 2020 by Suleman and Needhidasan [22], the authors chipped the electronic plastic waste (e-plastic) from printed circuit boards into sizes of a maximum of 20 mm and used it as a partial replacement of coarse aggregate in percentages ranging from 0% to 16.5%. The results showed a decrease in 28-day compressive strength for concrete with e-plastic starting at 6.5% replacement. A few other studies used PCBs to replace aggregate in concrete in either fine or coarse sizes [16]. A very limited number of studies pulverized PCBs into powder but mostly for purposes other than replacing cement in concrete. This study uses bare PCB powder to replace cement.

Powders can be used in concrete to partially replace cement as SCMs or as cement inert (filler). Powders can be considered SCMs and can be used to partially replace cement if they exhibit cementitious and/or pozzolanic properties. Pozzolan is defined by ASTM C219-25 [23] as “a siliceous or siliceous and aluminous material, which in itself possesses little or no cementitious value, but in a finely divided form and the presence of moisture, chemically reacts with Calcium Hydroxide at ordinary temperatures to form cementitious hydrates”. For SCM powders to have a reactivity comparable to cement, the size of the powder materials should be comparable to the grain size of the cement. A total of 95% of cement’s particles have a size of 45 microns.

On the other side, powders can be considered cement fillers if they have a particle size of less than 125 microns and can affect concrete on three levels. On the physical level, fillers improve particle packing and the compactness of concrete by filling the voids between cement particles. On the surface–chemical level, they enhance the cement hydration process by acting as nucleation sites to densify and homogenize the cement paste. On the chemical level, some cement fillers can react with Calcium Hydroxide and form cement gel. If they have pozzolanic reactivity, they can partially replace cement if no significant loss in strength is achieved [24].

Plastic powder by itself has neither siliceous, aluminous, nor pozzolanic properties and should not be expected to be used as an SCM to partially replace cement, nor it is expected to interact with cement paste at the physical level or surface–chemical level as a filler. Plastics are known for their hydrophobia phenomenon, which affects the interfacial transition zone between plastic particles and cementitious matrix [4,8]. This phenomenon is expected to inhibit the hydration and nucleation of the cement near plastic powder surfaces, preventing surface–chemical interaction. For this reason, PP was used in this study to replace fine aggregate and was not used in powder form to replace cement.

There is limited research work on utilizing plastic in concrete in powder form. Gesoglu et al. [25] used PVC waste powder in self-consolidated concrete (SCC) as a partial replacement of cement by mass. The results indicated a reduction in most mechanical properties measured in this study, with less brittle behavior for concrete containing plastic powder. Due to the difference in the specific gravity between the plastic powder and concrete constituents, a separation of the powder floating to the surface of the fresh concrete body was also observed.

In contrast, electronic fiberglass (e-fiberglass) waste was found to be an amorphous material, as indicated by the X-ray diffraction (XRD) technique. E-glass fibers with sizes of less than 75 µm have been found to have cementitious binding capabilities and can be considered SCMs [26]. For this reason, bare PCBs were ground in this study into powder form and used to partially replace cement.

Since glass is amorphous silica, it reacts in a high-PH solution to produce an alkali-silica reaction (ASR) gel. The ASR gel causes the expansion of concrete as the gel absorbs water. This expansion was found to generate internal stresses in the microstructure of the concrete [27]. Two factors were found to affect the use of glass in concrete: the percentage of replacement and the glass particle size. Smaller sizes of glass particles (smaller than 425 µm) were found to hydrate more actively, reducing the ASR effect [28], and cement replacement with less than 25% was found to increase the compressive strength of concrete when glass particles that had a size of less than 40 µm were used [27].

While the author in another study [29] investigated the mechanical performance of concrete incorporating different types of plastics, PP was used in this study because of its high resistance to the alkaline cementitious matrix solution. The alkalis in concrete were found to attack plastics embedded in concrete and caused the plastic to degrade over a long period. Different groups of plastics have shown different resistance to these alkalis under ambient conditions [30,31]. In a study [32], an improvement in the flexural strength of concrete reinforced by PET fibers was observed at 28 days and the improvement was no longer present after 150 days because PET degraded in the alkaline concrete environment.

This study aims to utilize post-consumer plastic and electronic waste (e-waste) in concrete to develop a plastic–concrete composite material that has good structural performance and contributes to the solution of the environmental problems associated with cement production and plastic waste. This process targets facilitating the removal of large volumes of end-of-life plastic products from landfills, and reducing the carbon footprint of concrete by partially replacing cement with bare printed circuit board (PCB) powder. In this research study, a post-consumer plastic product was ground into fine particles and e-waste was pulverized into powder, and each was used to produce a stable concrete mixture with desired mechanical properties and structural performance that can be considered in infrastructure applications.

## 2. Mechanical Processing

A significant portion of plastic products is not accepted by curbside recycling companies and goes to landfills and incineration. Reusing non-recyclable plastic products in concrete creates a market for their collection and use. Secondhand lawn chairs with plastic Type 5 (Polypropylene, PP) were collected for this study. The choice for this plastic type was dependent on the results of a previous research study [29]. The lawn chairs utilized were cut using a band saw into pieces with a maximum size of 5 in. × 5 in. (127 mm × 127 mm) to fit the granulator that was used to grind the plastic further. More details can be found in [33].

The Cincinnati Milacron mechanical granulator in the Integrated Systems Engineering (ISE) laboratory at the Ohio State University was used to grind the recycled plastic product to artificial fine aggregate with size of less than 4.76 mm (0.187 in.). The fineness modulus of the non-sieved PP particles directly collected from the granulator was found to be 3.85. PP particles were then sieved with a mechanical shaker as per ASTM C136/C136M-19 [34] and used to replace sand particles of the same size.

**Figure 1:**
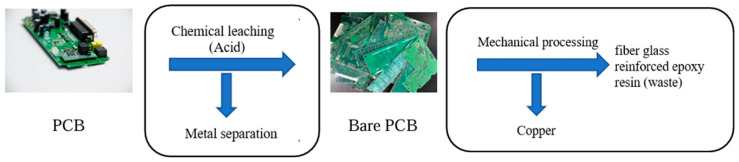
Metal-refining process for printed circuit boards [33].

On the other hand, a manual surface grinding tool was used to grind the bare PCBs into micro-sized powder, as further described in [33]. The bare PCBs used in this study were provided by Columbus MicroSystems, a company located in Columbus, OH, USA which provides technology recycling services. The PCBs used in this study were mostly from computers and medical devices. All metals and non-metals were removed from the exterior surfaces of the PCBs. Columbus Microsystems indicated that hydrochloric acid was used in the metal extraction process. The bare PCBs that are left after the metal extraction process contain layers of thin copper sheets integrated into epoxy resin and fiberglass, which were ground into powder in this study. This research aims to utilize the non-metallic components after the metal refining process of the PCBs. Figure 1 summarizes the refining process in PCBs.

**Figure 2:**
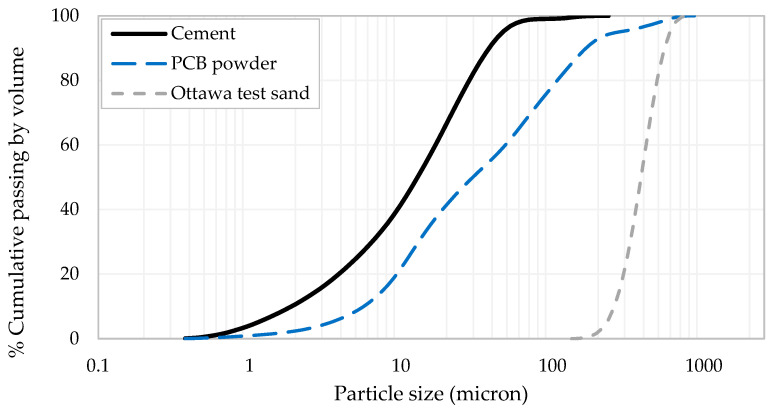
Particle size distribution for fine materials used in this study.

In this study, the particle size distribution for the PCB powder was evaluated using a laser diffraction analyzer located in the laboratories of the Department of Food, Agricultural and Biological Engineering at the Ohio State University. The PCB powder was found to have particles measuring less than 600 µm, and almost 60% of the powder had particle sizes of less than 45 µm (Figure 2).

The standard ASTM C1567-25 [35] was followed to provide a preliminary evaluation of the potential deleterious alkali-silica reaction (ASR) of the PCB powder when used in cement mortar. The PCB powder was used as a 10% replacement of cement by mass. The standard accelerated mortar bar test method (AMBT) was used to detect potential ASR within 16 days. Three specimens of standard cement mortar bars with a metal gage stud at each end were cast for testing ASR.

**Figure 3:**
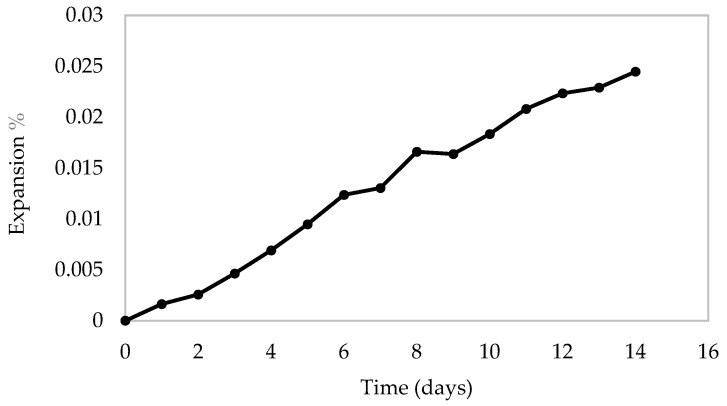
Measured percentage of expansion for mortar bars for ASR testing [33].

After the initial reading, the specimens were immersed in water, and the container was stored in an oven at 80 °C for 24 h. To determine the zero reading, the bars were removed one at a time from water and had their surface dried before determining the reading. Afterward, the specimens were stored in an oven at a temperature of 80 °C and immersed in a Sodium Hydroxide solution 103 (NaOH) for the rest of the 14 days of testing. Daily subsequent comparator readings of the specimens were collected (Figure 3). After 14 days of testing, expansion was found to be less than 0.1%. While the standard method requires measuring expansion for 16 days, the linear relationship in Figure 3 does not exceed 0.1% expansion at 16 days, indicating that the PCB powder is not reactive to the alkali solution of the cementitious matrix.

## 3. Physical Properties of the Materials Used in the Concrete Mixtures

For the fine aggregate used in this study and PP plastic particles ground to the size of sand, absorption was determined as per ASTM C128-22 [36]. ASTM C127-25 [37] was used to determine the absorption of the naturally rounded coarse aggregate used in concrete mixtures. To determine the specific gravity of the fine materials in this study, a pycnometer calibrated at one liter was used as per ASTM C128-22 [36]. To determine the specific gravity for powders including PCB powder, following the ASTM C188-25 [38] requirements, a Le Chatelier flask was used. To determine the specific gravity of the coarse aggregate used in the concrete mixtures, ASTM C127-25 [37] was used. Table 1 summarizes some physical properties of the materials used in this study.

Due to the complexity of bare PCBs in terms of components and composition, it is hard to separate their components for the purpose of determining their specific gravities. The high specific gravity of bare PCBs powder compared to PP particles can be justified due to the presence of copper and fiber glass with the epoxy resin. The typical specific gravity of epoxy resin is 1.2, copper typically has a specific gravity of 8.95, and for fiber glass, it is typically 2.55.

## 4. Mix Proportions of Concrete

Concrete was prepared according to a ratio of mass proportion of the cement to the fine aggregate to the coarse aggregate of 1:0.95:1.45. The concrete was designed and proportioned to achieve a compressive strength of 6000 psi (41.4 MPa). Since the specific gravity for the PCB powder was within the range of specific gravities of most pozzolanic materials used in concrete, bare PCB powder was used for 10% replacement of cement by mass. On the other hand, because of the relatively low specific gravity of PP particles compared to the crushed limestone fine aggregate, recycled PP plastic was used for 10% volumetric partial replacement of sand. PP particles were sieved with a mechanical shaker as per ASTM C136/C136M-19 [34] and used to replace sand particles of the same size. PP particles passed through No. 8 and retained on No. 16 were used for sand replacement. Table 2 shows the mix proportions for the materials used in the concrete mixtures.

To eliminate the workability effect on the results, a superplasticizer was added in equal dosages to the concrete mixtures, as suggested by the admixture manufacturer.

## 5. Methods for Testing and Assessment

The sections below describe the standard methods used to prepare and test concrete specimens and reinforced concrete (RC) beams, and summarize the results for the compressive strength development of concrete cylinders with cement and fine aggregate replacement compared to control cylinders. They also mention the results of the flexural testing of RC beams using replacements compared to control RC beams, and summarize the evaluation for the life cycle assessment (LCA) analysis that was used to estimate the environmental impact of general replacement of cement and fine aggregate in concrete.

### 5.1. Specimen Preparation and Testing

Concrete cylinders were prepared and tested to determine the effect of using PP particles and PCB powder on the strength development of concrete. ASTM C39/C39M-21 [39] was used to prepare and test 4 in. diameter and 8 in. height concrete cylinders for compressive strength. More details on the compressive strength testing are available in [33].

**Figure 4:**
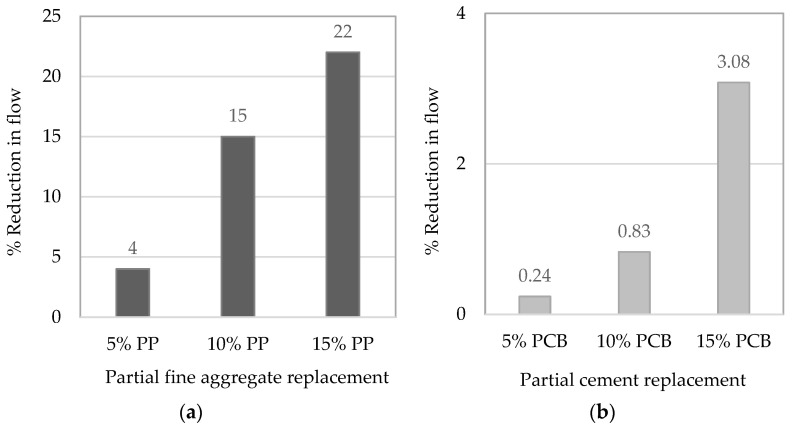
(**a**,**b**) Reduction in the average compressive strength at the age of 28 days for mortar cubes incorporating different types of plastics at different percentages of replacements.

Due to the difficulty of producing PP particles to replace sand particles of the same size and of pulverizing the bare PCBs into a powder, the flow of the mortar was tested in order to understand the effect of using plastic and e-waste on workability. The plastic fine aggregate was incorporated in proportions of 5%, 10%, and 15% by volume. However, the PCB powder was used as a filler (inert) to partially replace cement by mass in proportions of 5%, 10%, and 15%. ASTM C1437 [40] and ASTM C230 [41] were used for this purpose. Figure 4 shows the reduction in the average flow of fresh mortar incorporating different types of plastic particles at different percentages compared to the control mortar mix.

### 5.2. Compressive Strength Development

**Figure 5:**
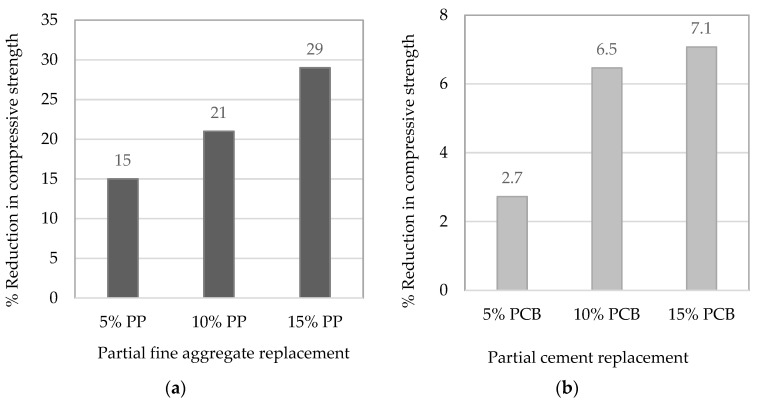
(**a**,**b**) Reduction in the average flow for fresh mortar incorporating different types of plastics at different percentages replacements.

Due to the difficulty of producing plastic particles of a certain size to replace fine aggregate and cement, compressive strength development of mortar with 2 in. size (50.08 mm) cube specimens was conducted for a different percentage of replacement, as per ASTM C109 [42]. The plastic fine aggregate was incorporated in proportions of 5%, 10%, and 15% by volume. However, the PCB powder was used as a filler (inert) to partially replace cement by mass in proportions of 5%, 10%, and 15%. Figure 5 shows the reduction in average compressive strength at the age of 28 days of mortar cubes incorporating various types of plastics at different percentages of replacement. Further details about mortar testing can be found in [29,33].

**Figure 6:**
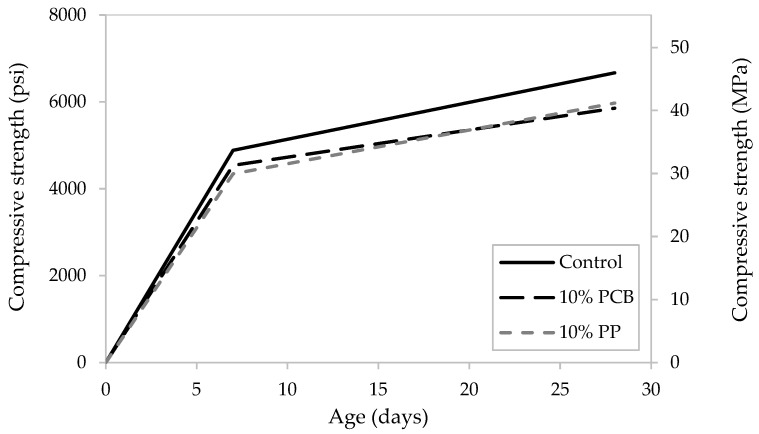
Measured average compressive strengths of control concrete cylinders compared to cylinders incorporating PP particles and PCB powder as 10% replacement at 7 and 28 days.

Table 3 summarizes the results of the compressive strength testing of the standard concrete cylinders. Five cylinders were tested to determine the average compressive strength of concrete at 28 days. The cylinders were demolded 24 h after casting and tested under uniaxial compressive strength at 7 and 28 days. A hydraulic compressive testing machine applying an axial load at a rate of 35 psi/s (0.241 MPa/s) was used. A drop in the compressive strength was observed for concrete incorporating 10% PP and 10% PCB (Figure 6). The choice to use 10% plastic replacement was to increase waste valorization without much reduction in compressive strength.

The reduction in compressive strength reported in Table 3 was calculated by subtracting the average compressive strength for control cylinders from the average compressive strength for cylinders with replacement and divided by the average compressive strength of the control cylinders.

### 5.3. Flexural Behavior of Reinforced Concrete Beams

**Figure 7:**
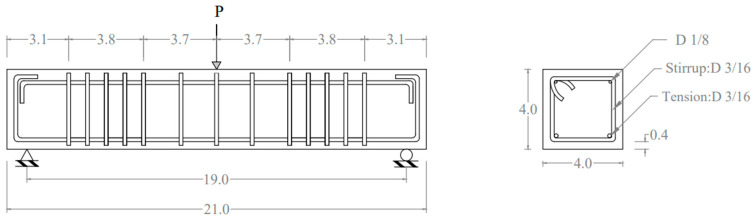
Details of the lab-scaled reinforced concrete beam; dimensions in inches (1 inch = 25.4 mm) [33].

To examine the behavior of concrete incorporating bare PCB powder and PP particles in structural applications, reinforced concrete (RC) beam specimens were designed and tested under flexural loading. The beams had a total length of 21 in. (533 mm), a span length of 19 in. (483 mm) and a 4 in. (102 mm) square cross-section (Figure 7). The shear span-to-effective-depth ratio was 3.43.

A total of six specimens were tested after 28 days from casting: two from each concrete mixture (control or no replacement, 10% PP particles replacing sand, and 10% PCB powder replacing cement). Reinforcement of the beams is detailed in Figure 7. Cold-rolled plain solid round low-carbon steel bars were used with a tested and recorded ultimate tensile strength of 95.7 ksi (660 MPa).

Due to the difficulty of pulverizing the bare PCBs into a powder form and of producing PP particles to replace sand particles of the same size, flexural testing was conducted using lab-scaled reinforced concrete beams. The research efforts reflect a preliminary structural study to examine the feasibility of using concrete incorporating plastics with different sizes in some infrastructure applications. As this study highlights maximizing waste valorization, the effect of the size of waste plastic incorporated in concrete on different LCA measures is discussed later in Section 5.4.

**Figure 8:**
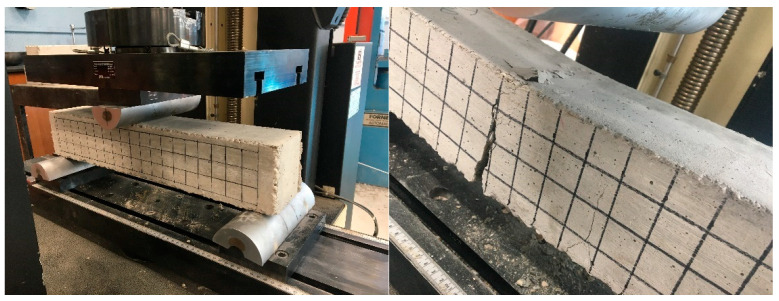
Flexural testing setup and crack propagation at the midspan.

The simply supported reinforced concrete beam specimens with hinge–roller supports at the ends were tested using an Instron universal testing machine that has a maximum load capacity of 30 kips (133.4 kN), as shown in Figure 8. The rate of vertical displacement used in testing at the midspan was 0.001 in./second (0.0254 mm/second).

**Figure 9:**
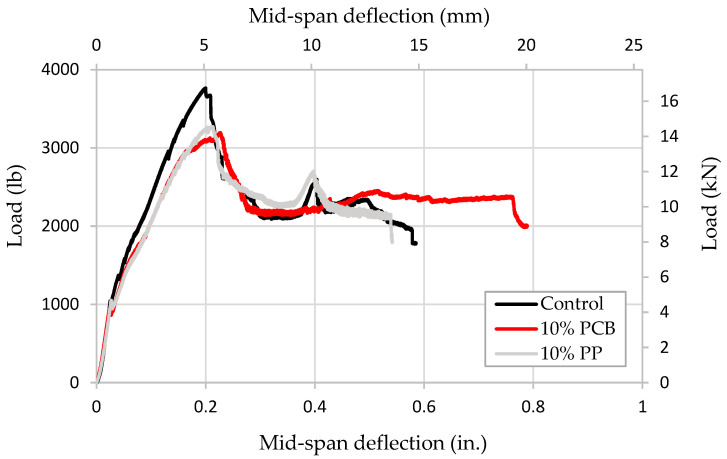
Average load—midspan deflection for reinforced concrete beams tested under flexure at age of 28 days.

The average load–midspan deflection curves for the reinforced concrete beams tested under flexural loading are shown in Figure 9. The main results for the reinforced concrete behavior extracted from the curves are summarized in Table 4. A reduction in the load capacity of the beams was observed when PCB powder and PP particles were incorporated into concrete compared to control specimens. On the other hand, the deformation capacity was observed to increase when the PCB powder was used to replace 10% of the cement, while the maximum displacement reduced when PP particles replaced 10% of the fine aggregate (Table 4). Δ*_u_* is the maximum deflection on the load–midspan deflection relationship. The stiffness was determined by finding the slope for the linear portion of the load–midspan deflection relationship. A more detailed discussion of the test results is available in [33].

The maximum load capacity of the RC beams incorporating the PCB powder was found to be the lowest, while the highest was recorded for control beams. The drop in maximum load capacity for the beams incorporating the PCB powder is explained due to utilizing the PCBs to replace cement in the concrete. On the other hand, maximum midspan deflection before failure was observed to be highest for beams incorporating PCB powder. On the contrary, the lowest displacement was observed in RC beams incorporating PP particles as a sand replacement, where the lack of bond between the plastic particles and the surrounding concrete more likely caused early microcracks to initiate at the surface of the plastic particles that propagated and reduced the displacement capacity at failure.

It is believed that a slight bond slip took place in some specimens before failure due to using plain steel reinforcing bars. However, the maximum deflection at maximum load capacity was found to be comparable and slightly differs for the specimens. The midspan deflection associated with the maximum load capacity was recorded as 0.2 in. (5.08 mm) for control beams, 0.215 in. (5.461 mm) for beams with PP particles, and 0.22 in. (5.588 mm) for beams with PCB powder. While typically deformed bars are used in reinforced concrete to prevent slipping, the maximum size for the reinforcing bars found in the market to produce beam capacities within the testing machine limitations was only that of plain bars.

The average initial stiffness, which is determined by the slope of the load–displacement diagrams, was found to be the largest for the control RC beams compared to RC beams incorporating the PCB powder or PP particles. Crack initiation and propagation were monitored by visual inspection during and after testing.

Table 5 shows that the ratio of $M_{exp}/M_{n}$ is greater than 1.0, which indicates that the strain compatibility method using ACI 318-25 [43] provisions for designing RC beams can potentially be employed to evaluate the flexural capacity, $M_{n}$, of RC beams with concrete incorporating plastic. Further testing utilizing large-scale beams is required to verify this result. Equation (1) was used to evaluate the experimental flexural capacity, $M_{exp},$ of the RC beams using the maximum measured load capacity, P. Equation (2) was used to calculate the flexural capacity of the beams using strain compatibility and the mechanical properties of the materials used in this study.

(1) $$M_{exp}=PL/4$$

(2) $$M_{n}=f_{y}A_{s}(d_{t}-a/2)$$

where L is the span length of the beam which is 19 in. (483 mm). $f_{y}$ is the yield tensile strength of the steel rebar, which is 60 ksi (414 MPa). $A_{s}$ is the total cross-sectional area of the steel rebars used on the tension side of the beam. $d_{t}$ is the distance from the centroid of the tension bars to the top of the beam section for the RC specimen, and a value of 3.53 in. (70 mm) for the RC specimen shown in Figure 7 was used. *a* is the depth of compression stress block and calculated following ACI 318-25 [43] as $a=A_{s}f_{y}/\beta_{1}f_{c}^{\prime }b$. $\beta_{1}$ is the stress block depth factor and calculated as $0.85-0.05(f_{c}^{\prime }-4000)/1000$(units is psi) and b is the width of the cross-section of the beam and equals 4 in. (102 mm) (Figure 7).

### 5.4. Comparison of Estimated Life Cycle Assessment Measures

A life cycle assessment (LCA) was conducted to compare the environmental impact related to the partial replacement of cement and fine aggregate in concrete. The main goal of the LCA study was to quantify the potential environmental impacts resulting from replacing 10% of the cement compared with replacing 10% of the sand in the production of 1.0 cubic yards of concrete. The software Athena Impact Estimator for Buildings version (5.4.0103) was used for this study using the mix proportions in Table 1. To justify zero (negligible) environmental impact for the replaced waste materials, the alternative material to replace cement or sand was assumed to be a waste and needed no further processing to be used in concrete.

**Figure 10:**
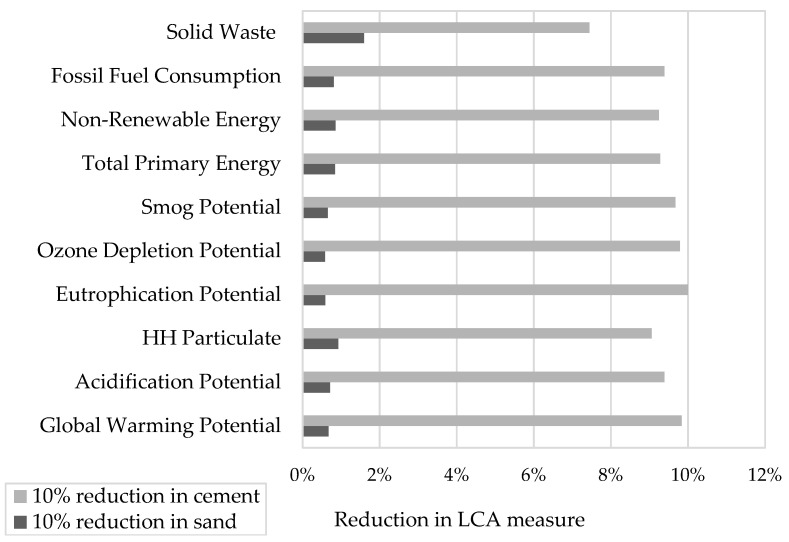
Comparison of the reduction in LCA measures when sand and cement reduction in concrete was applied [33].

The waste material to replace cement is considered to equal zero (negligible) in this study to overcome the lack of information in the LCA measures for the recycling processes needed to produce the material in a form that can be used in concrete. There are no current processes in the market to produce plastic aggregate with well-defined collecting, sorting, grounding, and sieving processes. As a quick comparison, the LCA measures might be approximate for producing both the limestone aggregates and the plastic aggregates in order to be used in concrete. Although, it is worth considering that utilizing plastics in concrete to replace natural aggregates can protect habitats and create a market for types and products of plastics that are non-recycled and end up in landfill or incinerated. Nevertheless, the environmental impact shown in Figure 10 shows a reduction of almost 1% for most of the LCA measures for fine aggregate replacement. On the other hand, if the electronics recycling facilities produce PCBs in a powder form for the purpose of refining metals, and this powder is used directly in concrete without the need to further processing, then the reduction in LCA measures is approximately 10% for most LCA measures, as shown in Figure 10. It is worth considering that this could be one of the best practices to bury PCBs that might contain residuals of hazardous materials: using these in concrete with low percentages of cement replacement considering the acceptable exposure limits. Typically, cement only makes up 10–15% of concrete.

The functional unit in the LCA was the production of 1.0 cubic yard (0.76 cubic meter) of concrete. The compressive strength of the concrete with both cement and fine aggregate replacements was assumed not to significantly differ at 28 days. The functional unit of 1.0 cubic yard (0.76 cubic meter) of concrete was approximated in Athena Impact Estimator software by defining a slab-on-grade assembly with a length of 10 ft (3 m), a width of 8.1 ft (2.5 m), and a thickness of 4 inches (102 mm).

The system boundary for the environmental assessment of the concrete in this study includes only the production phase (A1–A3), which is from the cradle to the end gate of the ready-mix concrete plant. Only the manufacturing in the production phase was included. The transportation effect was excluded. The boundaries of the environmental impacts of cement and aggregate were also considered from the cradle to the gate of their production plants. For all concrete mixes, chemical admixtures are excluded from LCA analysis due to the low relative contribution of the admixture to the environmental impact of concrete.

This experimental study reflects a single case of replacing cement with PCBs (10% by cement mass) and a single case of replacing sand with PP particles (10% by sand volume) in concrete specimens. Table 1 shows the mix proportion for the materials used in the concrete mix to produce 6000 psi (41.4 MPa) strength at 28 days and used in the LCA study as material consumption. It should be noted that using the PCB powder as cement replacement resulted in utilizing more mass of waste material than incorporating PP particles as a fine aggregate (see Table 1). The reason is the higher specific gravity of the PCB powder, which justifies replacement by mass, while volumetric replacement was considered for the plastic particles because of their low specific gravity compared to the specific gravity of the replaced sand. The use of PCBs to replace cement by mass increases waste valorization.

The LCA measures included in this study were Global Warming Potential (units in kg CO_2_ equivalent), Acidification Potential (units in kg SO_2_ eq), Human Health (HH) Particulate (units in kg PM2.5 eq), Eutrophication Potential (units in kg N eq), Ozone Depletion Potential (units in kg CFC-11 eq), Smog Potential (units in kg O_3_ eq), Depletion of Non-Renewable Energy (units in MJ), and Solid Waste (units in kg).

In this study, the PCB powder was used in specimens to replace 10% of cement, and PP particles were used as a 10% replacement for sand in other specimens. The cutback in cement content was found to have a significant reduction in the different LCA measures, approximately 10% except for the Solid Waste to Landfill effect, compared to the negligible effect of replacing sand, approximately 1%, as shown in Figure 10. More details of the LCA analysis can be found in [33].

## 6. Conclusions

This research investigated the effects of replacing cement and sand with post-consumer plastic and electronic waste products. The compressive strength of concrete, the flexural behavior of reinforced concrete beams, and LCA effects are examined. Based on the results of this study, the following conclusions can be drawn:

- When 10% of the cement content in concrete was replaced with PCB powder, a 12% reduction in the compressive strength was observed when concrete was tested at the age of 28 days;
- When 10% of the fine aggregate in concrete was replaced with PP particles, a 10.5% reduction in compressive strength was observed when concrete was tested at the age of 28 days;
- When the alkali-silica reaction (ASR) of the PCB powder was evaluated in the cement mortar, the expansion was found to be less than 0.1% after 14 days of testing, indicating that the PCB powder is not reactive to the alkali solution of the cementitious matrix;
- To evaluate the structural behavior of the concrete, including PP and PCB materials, reinforced concrete beams were tested under flexure. The maximum load capacity decreased by approximately 15% when PCB powder replaced cement by 10%, while the reduction was 13% when PP particles replaced fine aggregate. PCB powder reduced the cement content in the concrete;
- Control beams had the highest load capacity and stiffness. On the other hand, reinforced concrete beams with PP particles showed the lowest displacement capacity. PP particles are expected to create a separation in the concrete under loading due to the lack of bond between the plastic and the surrounding cementitious matrix;
- The strain compatibility method using ACI 318-25 [43] provisions for designing RC beams was found to potentially be employed to evaluate the flexural capacity of RC beams with concrete incorporating plastic. Further testing utilizing large-scale beams is required to verify this conclusion;
- An estimation of the environmental impact of replacing different constituents in concrete showed a significant reduction in the environmental impact when thermosets, such as PCB powder, replace cement, compared to when thermoplastics, such as PP particles, partially replace fine aggregate in concrete. Replacement of sand by 10% PP particles had a negligible reduction in environmental impact. On the other hand, the replacement of cement with PCB powder by 10% led to an approximate 10% reduction in the overall environmental impact.

This study concentrated on evaluating the mechanical and environmental performance of utilizing some plastic products in concrete in different forms (fine aggregate, powder). The performance can be further investigated using larger-scale structural elements to ascertain the compatibility with building codes. Different durability aspects other than ASR should also be evaluated. Considering that the PCB powder might contain some residual hazardous metals following the refining process, leaching of concrete with PCBs should be evaluated to ensure the acceptable exposure limits are not exceeded. The reactivity of the PCB powder as a pozzolan material and the microstructure of the concrete utilizing plastic can be further tested following ASTM C311/C311M-22 [44]. More accurate study for LCA would be feasible by evaluating the LCA measures for further processes needed to generate plastic waste in a form that can be used in concrete.

## Figures and Tables

**Table 1:** Specific gravity of different plastic waste and e-waste used in this study.

Plastic Product	Specific Gravity	Absorption
PP particles	0.997	-
Printed circuit boards powder	2.6	-
Natural rounded coarse aggregate No. 89	2.5	2.5%
Crushed limestone fine aggregate	2.4	5%

**Table 2:** Mix proportions for concrete mixtures.

	Powder or Plastic Content, lb/Cubic Yard (kg/m^3^)	Crushed Limestone Sand, lb/Cubic Yard (kg/m^3^)	Natural Coarse Aggregate, lb/Cubic Yard (kg/m^3^)	Cement, lb/Cubic Yard (kg/m^3^)	Water, lb/Cubic Yard (kg/m^3^)
Control	-	930 (551.7)	1410 (836.5)	975 (578.4)	472 (280)
10% PP	39 (23.1)	837 (496.6)	1410 (836.5)	975 (578.4)	472 (280)
10% PCB	97 (57.5)	930 (551.7)	1410 (836.5)	878 (520.9)	472 (280)

**Table 3:** Average measured compressive strengths at the age of 28 days for control concrete cylinders and cylinders with 10% replacement incorporating PP particles and PCB powder.

	Average Compressive Strength, $f_{c}^{\prime }$, psi (MPa)	Reduction in Compressive Strength	Standard Deviation, psi (MPa)
Control	6671 (46)	-	88 (0.607)
10% PP	5973 (41.2)	10.5%	110 (0.758)
10% PCB	5854 (40.4)	12%	83 (0.572)

**Table 4:** Summary of average stiffness, load capacity, and midspan deflection of the reinforced concrete beams.

	Load Capacity, *P,* lb (kN)	Relative Load Capacity	Stiffness, kips/in. (kN/mm)	Relative Stiffness	$\Delta_{u}$, in. (mm)	Relative $\Delta_{u}$
Control	3765 (16.7)	1	52.4 (9.18)	1	0.585 (14.9)	1
10% PP	3271 (14.6)	0.87	45.4 (7.95)	0.87	0.542 (13.8)	0.93
10% PCB	3195 (14.2)	0.85	46.6 (8.16)	0.89	0.789 (20.0)	1.35

**Table 5:** Summary of compatibility of flexural behavior of reinforced concrete beams with ACI 318-25 [43] provisions.

	*P*, lb (kN)	Average Experimental $f_{c}^{\prime }$, psi (MPa)	$M_{exp}$, kips-in. (kN.m)	$M_{n}$, kips-in. (kN.m)	$M_{exp}/M_{n}$
Control	3765 (16.7)	6671 (46)	17.88 (2.02)	11.41 (1.290)	1.57
10% PP	3271 (14.6)	5973 (41.2)	15.54 (1.76)	11.39 (1.287)	1.36
10% PCB	3195 (14.2)	5854 (40.4)	15.18 (1.72)	11.39 (1.287)	1.33

## Data Availability

The original contributions presented in this study are included in the article. Further inquiries can be directed to the corresponding author.

## Abbreviations

The following abbreviations are used in this manuscript:

PCB	Printed Circuit Board
PET	Polyethylene terephthalate
HDPE	High-Density Polyethylene
PVC	Polyvinyl Chloride
LDPE	Low-Density Polyethylene
PP	Polypropylene
PS	Polystyrene
ABS	Acrylonitrile Butadiene Styrene
MSW	Municipal Solid Waste
ITZ	Interfacial Transition Zone
SCM	Supplementary Cementitious Material
